# The Buffer Effect of Therapy Dog Exposure on Stress Reactivity in Undergraduate Students

**DOI:** 10.3390/ijerph14070707

**Published:** 2017-06-30

**Authors:** Alexandra J. Fiocco, Anastasia M. Hunse

**Affiliations:** Department of Psychology, Institute for Stress and Wellbeing Research, Ryerson University, 350 Victoria Street, Toronto, ON M5B 2K3, Canada; anastasia.hunse@gmail.com

**Keywords:** stress, therapy dog, intervention, human-animal interaction

## Abstract

Stress is an insidious health risk that is commonly reported among university students. While research suggests that dog exposure may facilitate recovery from a stress response, little is known about the buffer effect of dog exposure on the stress response to a future stressor. This study examined whether interaction with a therapy dog could reduce the strength of the physiological stress response when exposed to a subsequent stressor. Sixty-one university students were randomly assigned to either a therapy dog (TD, *n* = 31) or a no-dog control (C, *n* = 30) group. The stress response was measured by electrodermal activity (EDA) in response to the Paced Auditory Serial Addition Test (PASAT). Participants also completed questionnaires that assessed pet attitude, general stress levels, and affect. Analyses of covariance (ANCOVAs) showed that increase in EDA was significantly more pronounced in the C group than in the TD group (*p* < 0.01). Pet attitudes did not modulate the buffer effect of therapy dog exposure. Results suggest that therapy dog exposure may buffer the stress response in university students, which has implications for the promotion of a viable stress management program on university campuses.

## 1. Introduction

Stress is an insidious pathophysiological mechanism that underlies a number of health-related disorders, both physical and psychological in nature [1,2]. Both affective and physiological stress is particularly pervasive in undergraduate students and has been linked to a number of negative health outcomes [3,4]. Further, it has been shown that students are commonly unknowledgeable of viable stress reduction options available to them on or off of campus [5].

The negative effect of stress on physical and psychological health outcomes in university and college students has been well documented. The transition into university can be associated with a number of perceived stressors, including: multiple examinations, concerns about grades, financial difficulty, and interpersonal or relationship stressors [4]. Heightened levels of stress are pervasive in the undergraduate student population and have been linked to the development of overall physical illness [6], depression [3,7], and anxiety [3]. Increased blood pressure has also been found to correlate with stress in the undergraduate population, both of which significantly increase during times of academic study [8,9].

In a 2013 survey that gathered information from 30,000 students at 30 Universities, the American College Health Association (ACHA) found that 40% of students had not received information about stress reduction from their university, while 74% were interested in receiving information on stress reduction. The same survey reported that 45% of students rated their stress as above average and 12% indicated that they were under tremendous stress [5]. In the ACHA Fall 2016 survey, completed by 36,385 students, when questioned which factors had a negative impact on their academic performance, 32.2% of students reported general stress, 24.9% reported anxiety, 15.4% reported depression and 20.6% reported sleep difficulties [10]. Further, 82.6% of students reported feeling exhausted, 60.8% felt overwhelmed by anxiety, 60.6% felt lonely, 50.9% reported feeling that things were hopeless, and 10.4% seriously considered suicide [10]. There has also been a notable rise in stress-related health ailments between 2013 and 2016; including a 50% increase in anxiety, 47% increase in depression, and a 47% increase in suicide attempts [10]. Together, these reports highlight the need for an effective, timely, and accessible source of stress relief for students on campus. One such source may exist in the therapy dog programs that have been increasing in popularity on university campuses.

A plethora of research suggests that pet ownership is significantly associated with wellbeing in Western, European, and Eastern countries (for review, see [11]). Research shows that animal assisted therapy produces a number of benefits in clinical populations, including improved focus and awareness in children with pervasive development disorders [12], increased language use in children with autism [13], decreased stress physiology within a social context in children with autism [14], increased social interactions in patients with dementia [15], and decreased depression in institutionalized patients [16]. One hour of interaction with a therapy dog has been shown to decrease self-report ratings of depression, anxiety, and pain in patients admitted to a mental health hospital [17]. Further, a study that examined residents of a nursing home found that weekly 15-minute interactions with a therapy dog led to an improvement in mood [18,19].

In non-patient populations, exposure to a therapy dog is associated with an increase in sense of well-being and pleasure [20], and is also found to be associated with greater decreases in endocrine and cardiovascular reactivity following stress exposure [21]. A study conducted by Odendaal and Meintjes [22] found that as little as five minutes of interaction with a therapy dog lowered cortisol levels and blood pressure compared to pre-test levels in healthy adults. Additionally, a study conducted by Barker et al. [23] demonstrated that a five-minute interaction with a therapy dog lowered serum and salivary cortisol levels in healthcare professionals compared with baseline levels. Presence of a therapy dog may further reduce cardiovascular arousal while engaged in a stressful situation [24,25].

While previous research suggests that dog exposure may facilitate recovery from a stress response, few studies have explored whether prior interaction with a therapy dog before stress exposure can act as a buffer to subsequent stress responsivity. Allen et al. [26], examined the effect of a human versus animal companion on stress reactivity in response to an arithmetic challenge in 45 female dog owners, aged 27 to 55 years of age, who were self-described as “lovers of dogs”. Women were tested in their home and were randomly assigned to one of three conditions: a no companion condition, a human companion condition, and a pet companion condition. Results showed that women assigned to the pet companion condition displayed lower stress reactivity, as measured by the galvanic stress response (i.e., electrodermal activity, EDA) and systolic blood pressure, compared with the human companion and no companion condition [26]. This study suggests that the presence of a pet during stress exposure serves as a buffer to the stress response. Although research suggests that presence of a pet may reduce stress physiology during stress exposure [26,27,28], these studies have focused on participants who are pet owners, using the participants’ personal pet in the protocol. In these study designs, participants underwent a mental stress induction protocol in the comfort of their home while their pet roamed freely. There is a paucity of research that has examined the effect of brief therapy dog exposure on subsequent stress responsivity to a stress induction protocol during which the therapy dog is not present. This research question is important in the context of university and college students who may not have access to a personal pet, but may have brief access through programs such as a therapy dog program. Indeed there is a growing interest among students for the use of therapy dogs as a method of stress reduction [29].

To this end, the present study aimed to determine whether interaction with a therapy dog impacts the physiological stress response in the face of a subsequent stressor, and whether attitude towards pets might moderate this effect. Based on previous literature, it was hypothesized that interaction with a therapy dog would be associated with lower levels of physiological stress reactivity, as measured by EDA. It was further predicted that the buffer effect would be enhanced by high self-report positive pet attitudes.

## 2. Materials and Methods

### 2.1. Participants

Sixty-one university students, with a mean age of 21.02 (*SD* = 5.503, range 18–47) were randomized to either the therapy dog (TD) group (*n* = 31) or the control group (*n* = 30). There were 14 male participants and 47 female participants. Exclusion criteria for the study included fear and/or allergies to dogs. Participants were invited to the Institute for Stress and Wellbeing Research where they signed an informed consent form before undergoing the study protocol. Participants were naïve to the true purpose of the study, and were fully debriefed at study completion. This study was carried out following the rules of the Declaration of Helsinki of 1975. This study was granted ethics approval by the Ryerson University Research Ethics Board (2014-308).

### 2.2. Procedure

Participants were randomized to either a Therapy Dog (TD) or no-dog Control (C) group, using a blocked design randomizing software called Research Randomizer [30]. Participants were unaware of which group they had been assigned to prior to their appointment, and were informed upon enrollment that group assignment was random. Each participant was tested in a private testing room.

**Figure 1:**
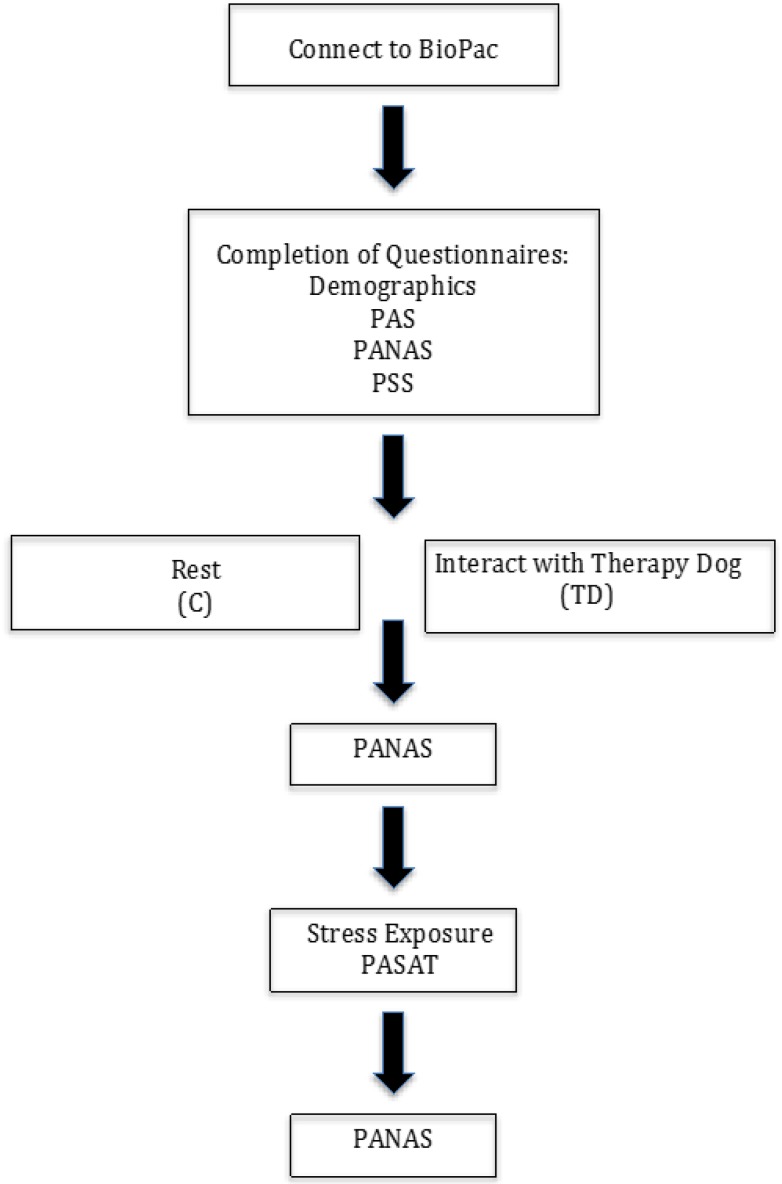
Flowchart of the study procedure. PAS: Pet Attitude Scale; PANAS: Positive and Negative Affect Scale; PASAT: Paced Auditory Serial Attention Task; PSS: Perceived Stress Scale.

Once consent had been collected, participants’ finger-tips were fitted with a Biopac galvanic skin response amplifier. Following a five-minute baseline rest period, participants in both groups completed questionnaires that gathered information about demographics, perceived stress, and pet attitudes. Participants in the C group were then told to relax in a seated position for 10 min. Participants in the TD dog were asked to remain seated while a therapy dog was brought to sit beside them. TD group participants were informed that they could interact with the therapy dog in a seated position for 10 min, after which time the therapy dog would be removed from the room. After this 10-min experimental period (i.e., no dog vs. dog) participants were asked to complete an affect questionnaire. Upon completion, participants were given instructions about how to complete the Paced Auditory Serial Attention Task (PASAT). Upon completion of the PASAT, participants were asked to fill out a second affect questionnaire before being detached from the Biopac system and undergoing the debriefing process. See Figure 1 for a flowchart of the procedure.

### 2.3. Measures

#### 2.3.1. Stress Induction

*Paced Auditory Serial Addition Task (PASAT)* [31]. The PASAT is a computerized audio task that assesses attentional processing, immediate memory, and attention [32]. It has been also been shown to reliably induce a stress response [33]. In order to induce participants’ perceived threat to ego and increase their stress response, participants were told that their performance was going to be monitored by the researcher and was being compared with other students. Completion of the PASAT included listening to a standardized audio recording of single digits being presented every 3 s, with the participant expected to add each digit to the one immediately prior to it and input it at increasing speed. The PASAT took approximately 8 min to complete.

#### 2.3.2. Stress Buffer

*St. John Ambulance Therapy Program (Toronto, ON, Canada).* The present study used ten therapy dogs recruited from the St. John Ambulance organization. Dogs ranged in age and size. Breed of dog included an Irish Setter, Schnoodle, Miniature Poodles, Greyhound, King Charles Spaniel, Golden Retrievers, and Australian Cattle Dog. All dogs were certified by the organization, and as such had certified good health, hygiene, and had been trained to interact with people in a docile manner. Participants in the TD group interacted with the therapy dog for ten min before stress induction.

#### 2.3.3. Physiological Stress Measures

*Galvanic Skin Response.* To monitor the physiological stress response, participants’ electrodermal (EDA) activity was measured throughout the entire testing process by a trained researcher using the Biopac EDA100C (Biopac Systems Canada Inc., Montreal, PQ, Canada). The EDA100C measures skin conductance levels as they increase when an individual is under stress, and is an efficient and effective means of measuring stress reactivity [34].

#### 2.3.4. Questionnaires

*General Perceived Stress* [35]. The Perceived Stress Scale (PSS) is a measure that determines the degree to which an individual perceives their life as stressful in the past month. The PSS contains 14 items on a 5-point scale with the following response options: never, almost never, sometimes, fairly often, and very often. The PSS has been shown to have excellent reliability and validity [36,37]. Higher scores on the PSS indicate greater perceived stress.

*Positive and Negative Affect* [38]. The Positive and Negative Affect Schedule (PANAS) is a measure that uses a 5-point scale, from “Not at all” to “Extremely”, to determine the range of feelings for 20 different emotions, including 10 positive (e.g., Interested, Excited, Enthusiastic) and 10 negative emotion (e.g., Irritable, Distressed, Nervous). The PANAS measures an individual’s emotions in the moment and is a way to determine change in affect after a stimulus is administered [38], Clark, & Tellegen, 1988). It has been shown to possess cross-sample stability, internal reliability, temporal stability, cross-cultural invariance, and convergent and criterion-related validity [39]. In the current study, the PANAS measured self-reported affect before intervention, after intervention, and following stress exposure. The scale provides a total score for positive affect (PA) and negative affect (NA), with higher scores indicating higher endorsement of the emotion.

*Pet Attitudes* [40]. The Pet Attitude Scale (PAS) measures participant’s attitudes towards pets. It is most commonly used to evaluate the interaction between testing results and people’s pre-existing attitudes towards pets. The PAS contains 18 items on a 7-point scale with response options including strongly disagree, disagree, slightly disagree, unsure, slightly agree, agree, and strongly agree. Higher score on the PAS indicates more positive attitude towards pets. The PAS is a viable choice to explore the relationship between participants’ physiological responses to the stressor and their pre-existing attitude towards pets, and has been shown to have high reliability [41].

### 2.4. Statistical Analyses

Data were assessed for assumptions of normality and outliers. One-way analyses of variance (ANOVA) and chi-square analyses were conducted to assess for between-group differences at baseline. Baseline factors that significantly differed between groups (*p* < 0.05) were controlled for in subsequent analyses. To address the study hypotheses, EDA and positive and negative affect levels were converted into change scores by subtracting the mean levels at baseline from the mean levels during the stressor. One-way ANOVAs were conducted on the change scores, controlling for covariates and baseline physiology scores. To assess the moderating role of pet attitude, a median split was conducted to create high and low categories for Pet Attitude. Interaction terms were then entered into the model (i.e., Group × Pet Attitude). Subgroup analysis was then conducted within the TD group to assess for moderation of pet attitude on therapy do buffer effect. All analyses were conducted using SPSS Statistics 22 (IBM Corp., North Castle, NY, USA) and results were considered significant at *p* < 0.05.

## 3. Results

### 3.1. Participant Characteristics

Recruitment for this study was conducted from January–February 2015. Overall, participants had a mean age of 21.02 years (range = 18–47). Analyses of variance and chi-square analyses showed that groups did not significantly differ on any of the baseline variables. Table 1 summarizes detailed sample characteristics and baseline measures.

### 3.2. Effect of Therapy Dog Exposure on Affective Reactivity

**Figure 2:**
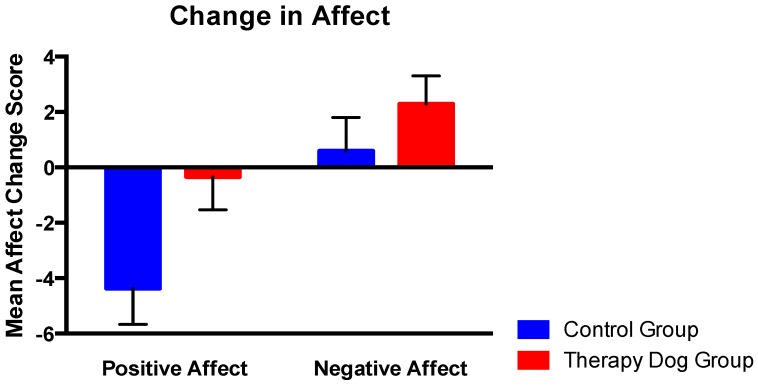
Buffer effect of therapy dog exposure on positive and negative affect.

Controlling for baseline affect, ANCOVA did not reveal a significant Group effect on change in positive affect, *F*(1, 61) = 3.17, *p* = 0.08. Although not statistically significant, there was a trend for positive affect to decrease more in the C group (*M*C = −4.37, *SD* = 7.15) compared to the TD group (*M*TD = −0.35, *SD* = 6.656) in response to the PASAT. Controlling for baseline affect, ANCOVA did not reveal a significant Group effect on change in negative affect *F*(1, 60) = 0.26, *p* = 0.61. Although not statistically significant, the mean change in negative affect was smaller in the C group (*M*C = 0.60, *SD* = 6.10) than the TD group (*M*TD = 2.29, *SD* = 5.62), see Figure 2.

### 3.3. Effect of Therapy Dog Exposure on Physiological Reactivity

**Figure 3:**
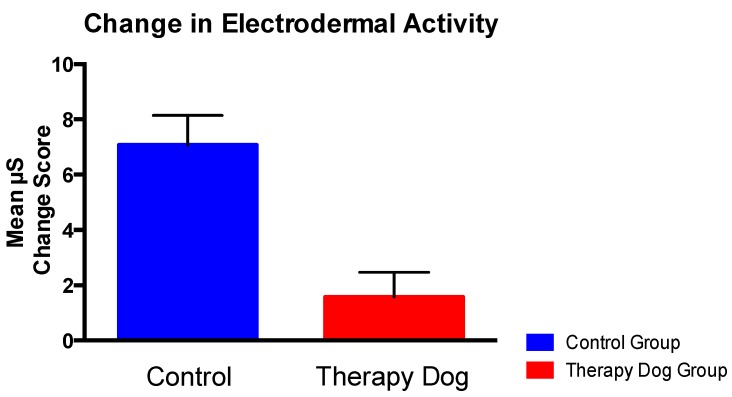
Buffer effect of therapy dog exposure on mean electrodermal activity.

Controlling for baseline EDA, ANCOVA showed a significant effect of Group on EDA change from baseline to post-stress, *F*(1, 59) = 15.24, *p* < 0.00. Specifically, the TD group showed a lower increase in EDA in response to the PASAT compared with the C group (*M*TD = 1.58, *SD* = 4.93 vs. *M*C = 7.07, *SD* = 5.81). See Figure 3.

### 3.4. Moderating Role of Pet Attitude in the Relationship between Therapy Dog Exposure and Stress Reactivity

ANOVA revealed no significant interaction between Group and Pet Attitude on change in EDA, *F*(1, 60) = 0.31, *p* = 0.31. Subgroup analysis restricted to TD participants showed no effect of High vs. Low Pet Attitudes on EDA, *F*(1, 31) = 0.41, *p* = 0.53. These results were confirmed by regression analyses.

## 4. Discussion

To date, a wealth of research has shown that pet ownership and therapy dog exposure can have tremendous beneficial effects for persons of all ages [11]. Research has shown that the presence of one’s pet during stress induction may buffer physiological reactivity [26,28]. Previous research also shows that exposure to a therapy dog can have beneficial effects in patient populations and can modulate physiological indices of stress such as blood pressure and cortisol [21]. This study adds to the wealth of knowledge in this area as it is the first study to show that interacting with a therapy dog for as little as 10 min may significantly buffer the stress response to a subsequent stressor.

Although the current findings are limited to university students, it is imperative to examine this population as they commonly report heightened levels of stress. Indeed, high stress levels in this population have been shown to associate with poor psychological outcomes, including increased levels of depression and anxiety [3]. Unsurprisingly, a large number of undergraduate students report that they desire access and information about effective stress reduction methods available on campus [5].

In accordance with the study hypothesis, therapy dog exposure significantly buffered the stress response, as measured by EDA. This is the first study to show that interacting with a therapy dog can dampen physiological reactivity in response to a future stressor, when the therapy dog is not present. In contrast, although a trending result was found for positive affect, the current study did not find therapy dog exposure to significantly influence self-reported affect in response to a stressor. Although null findings were not predicted, the contrast in findings between the physiological data and self-report data is not surprising. Indeed, it is well documented that objective measures of stress are not highly correlated with self-report measures [42]. Self-report measures may be affected by a number of extraneous variables, including personality and response bias. Accordingly, previous studies using affect as an outcome variable have been mixed; therapy dog exposure has been found to associate with increased feelings of calmness [43] and decreased depressive symptoms [44]. However, studies have also shown that interaction with a therapy dog can result in no change in dysphoria, positive affect [45], or mood in general [46]. Although not statistically significant, it is worth noting that participants exposed to a therapy dog reported a smaller decrease in positive affect after completion of the stress task than participants in the control group, suggesting that a recent interaction with a therapy dog may impact affective reactions to subsequent stress. The ability to mitigate the impact of stress on positive affect has intriguing implications, particularly since positive affect is associated with increased social engagement [47] and subjective wellbeing [48]. With respect to negative affect, while participants exposed to the therapy dog tended to display a greater increase in negative affect compared with participants in the control condition, it is important to note that participants in the control group reported a slightly higher mean negative affect at baseline. Therefore, these findings may be explained by a ceiling effect with respect to possible change in negative affect following the stressor.

Surprisingly, pet attitude did not moderate the stress-buffer effect of therapy dog exposure. Although this area is relatively unexplored in the literature, this finding differs from one study conducted in 1985 that found a significant positive correlation between positive pet attitudes and reduction in mean arterial pressure, systolic pressure, and diastolic pressure while petting a dog at rest [49]. The lack of significant findings regarding the relationship between pet attitude and stress reactivity in the present study may be explained by the characteristics of the current sample. The mean score for pet attitude was relatively high overall (*M* = 103.86). This indicates that a larger sample is needed to explore this relationship, where there is a wider distribution of participants scoring low on positive pet attitudes and those scoring high on positive pet attitudes. However, in the context of a university sample, the fact that a majority of students display a positive attitude towards pets may suggest that therapy dog exposure within the university is a viable stress-management program for undergraduates.

Although the current findings are promising, the study was not without limitations. The largest limitation was the relatively small sample size. Although there were sufficient participants per group to detect a significant difference in EDA, the sample may not have been large enough to assess the moderating role of pet attitudes. However, even with a larger sample size, the challenge of selection bias would likely remain. That is, students who dislike or fear dogs will likely choose not to participate in this research. This challenge may be addressed using more ecologically valid research designs, including the assessment of students who attend therapy dog sessions at their university. Another study limitation was that the majority of the sample was female. This is a consistent challenge with research, and is similar to previous studies that have largely based their findings on female participants (e.g., [26]). Given the importance of sex and gender differences in stress physiology [50], it is important to examine whether the buffer effect of pet therapy is moderated by gender or sex.

Additional research is needed to determine how pet attitudes and history of pet ownership moderates the beneficial effects of pet therapy on stress physiology and health outcomes. Further, research examining the longevity of the stress buffer effect is needed to provide more insight into how long individuals can benefit from therapy dog exposure.

Currently, there is a growing amount of interest in the student population for the use of therapy dogs as a method of stress reduction [29]. Qualitative reports suggest that therapy dog programs may decrease stress among university students who experience stress [51]. However, these programs require systematic evaluation to assess how therapy dog programs are helping student populations. We hope that this study will encourage additional research, assessing therapy dog exposure as a stress buffer in different populations that report relatively high levels of stress, including a more diverse group of university and college students from low socio-economic communities.

## 5. Conclusions

This study provides support for the use of therapy dog exposure as a viable stress-management tool to decrease stress physiology in undergraduate students, a segment of the population that displays increasing negative stress-related physical, mental and emotional health outcomes.

## Figures and Tables

**Table 1:** Baseline characteristics of participants.

Characteristic	Therapy Dog (*n* = 31)	Control (*n* = 30)	*p*-Value
	Mean (SD) or N (%)	Mean (SD) or N (%)	
Age, years	20.13 (3.364)	21.93 (7.017)	0.20
Female, N (%)	24 (39.3%)	23 (37.7%)	0.94
Ethnicity, N (%)			0.33
Black/African American	4 (6.6%)	1 (1.6%)	
White/Caucasian	10 (16.4%)	13 (21.3%)	
Other	17 (27.9%)	16 (26.2%)	
PAS	105.35 (14.87)	102.37 (17.83)	0.48
PSS	28 (8.34)	26.53 (6.25)	0.44
PANAS			
Positive	26.52 (6.51)	30 (7.31)	0.05
Negative	14.29 (3.37)	17.03 (6.87)	0.05

PAS: Pet Attitude Schedule; PSS: Perceived Stress Scale; PANAS: Positive and Negative Affect Schedule; EDA: Electrodermal Activity.
